# NutriA-Senior, a Mobile App to Enhance Nutritional Awareness in Older Adults: Design and Pilot Usability Study

**DOI:** 10.2196/89551

**Published:** 2026-11-12

**Authors:** Pietro Bosoni, Alessandra Vincenti, Federica Bolpagni, Cristiana Larizza, Nagaia Madini, Giovanna Nicora, Daniele Pala, Silvana Quaglini, Cecilia Ricciardi Rizzo, Lucia Sacchi, Sara Santero, Giulia Viroli, Hellas Cena, Giordano Lanzola

**Affiliations:** 1 Department of Electrical, Computer and Biomedical Engineering, University of Pavia, Via Ferrata 5, Pavia, 27100, Italy; 2 Laboratory of Dietetics and Clinical Nutrition, Department of Public Health, Experimental and Forensic Medicine, University of Pavia, Pavia, Italy; 3 Unit of Clinical Nutrition, Istituti Clinici Scientifici Maugeri, Pavia, Lombardy, Italy; 4 National Biodiversity Future Center, Palermo, Italy; 5 Department of Earth and Marine Sciences, University of Palermo, Palermo, Italy; 6 Department of Management, Information and Production Engineering, University of Bergamo, Bergamo, Italy

**Keywords:** mHealth, smartphone, older adults, healthy ageing, malnutrition, sarcopenia, patient education, questionnaires

## Abstract

**Background:**

Mobile health is emerging as a ubiquitous paradigm for delivering health care interventions when self-management is feasible. However, interventions targeting older or frail participants should account for age-related cognitive, visual, and physical needs to optimize usability and long-term retention.

**Objective:**

This paper describes the rationale, design, and implementation of Nutritional App-Senior (NutriA-S), a theory-informed mobile app developed to enhance nutritional education in older adults with sarcopenia or probable sarcopenia. Moreover, we present the results of a usability study conducted in a cohort of age-matched healthy volunteers.

**Methods:**

NutriA-S was designed as a self-instructional app built on the Moodle e-learning platform. It includes 24 progression modules containing interactive quizzes and associated informational items aimed at engaging users by sustaining attention and improving nutritional knowledge. A 4-week pilot usability study was conducted with 35 healthy volunteers aged 65 to 85 years, recruited through flyers posted in 2 outpatient settings. After recruitment, participants used the app remotely at home with telemedicine-based monitoring and support. At the end of the study, usability was quantitatively assessed using the Italian version of the standard System Usability Scale (SUS) and the User Version of the Mobile Application Rating Scale (uMARS).

**Results:**

The SUS questionnaire yielded a median total score of 80 out of 100 (IQR 55.00-92.50), indicating good perceived usability, with more than half of the participants rating the app as acceptable (above the threshold of 68). The overall median uMARS score was 4.083 (IQR 3.68-4.30), with the functionality and information domains achieving the highest median scores of 4.5 (with IQRs of 4.00-4.50 and 4.13-4.75, respectively), confirming that older adults perceived the tool as technically reliable and highly credible.

**Conclusions:**

This pilot usability study shows encouraging results, suggesting that NutriA-S is a highly usable, intuitive, and credible digital health tool for older adults. The identified limitations in items like customizability, willingness to pay, and willingness to use the app frequently, which received lower scores, will guide app refinements before deploying NutriA-S in an upcoming randomized controlled trial for older adults with sarcopenia or probable sarcopenia.

## Introduction

The ubiquitous diffusion of mobile devices hosting dedicated apps is reshaping the way in which almost every business is accomplished, including the delivery of health care, a concept referred to as mobile health (mHealth) [1,2]. This paradigm shift in the health care area finds its foundation in the potential of mHealth to assist patients in the self-management of diseases, thereby ensuring independent living for older adults [3], even in the presence of declines in their intrinsic capacity [4].

The potential of mHealth extends to the field of nutrition, where it is seen as a way of promoting changes in habits and lifestyle, as witnessed by the large number of nutrition-related apps available on the Google Play Store [5]. From a strictly clinical point of view, mHealth is a fundamental component of disseminating nutrition education as part of a regular treatment plan [6-8]. In fact, mHealth apps enable patients to manage their own health and support nutrition-related behavior change and outcomes, especially when used under the remote guidance of a clinician [8,9]. Nutritional interventions delivered through mHealth are also becoming increasingly popular among older adults [10].

Several health-promoting mobile interventions have been developed, including those concerning healthier diets [10]. This approach has shown potential in improving the outcomes of older participants affected by chronic illnesses [11]. mHealth facilitates treatment adherence and behavior change, improves the communication between patients and clinical staff, while also fostering self-monitoring. With the aging of the population, resorting to this technology will be unavoidable to increase the chance of spending the later stages of life in good health.

Nevertheless, despite this growing trend, most mHealth interventions are not specifically tailored for older and frail populations, frequently lack theory-based strategies, and remain largely underevaluated in clinical trials [12]. Moreover, since many older individuals may have lower levels of digital literacy compared with younger participants, systems that are simply repurposed for this population often overlook the unique cognitive, physical, and usability needs specific to older adults [13].

Given these criticalities, user acceptance is considered a key factor for the success of any mHealth nutritional intervention. Existing literature confirms that nutritional interventions delivered through digital devices targeting middle-aged and older participants face adoption barriers, even when the target population has adequate digital literacy [14]. Identifying and mitigating the underlying causes of these barriers is therefore essential for achieving the desired interventional impact.

This work was conducted within the Nutritional App (NutriA) project, which is part of ON Foods, the Italian research and innovation network for sustainable food and nutrition [15]. This project developed a framework for digital nutrition interventions, including NutriA-IBD (NutriA–inflammatory bowel disease), an app supporting people with IBD [16], and NutriA-Junior (originally introduced as V-Care) to support lifestyle improvement in children with obesity [17]. In synergy with the Sustainable Eating Patterns to Limit Malnutrition in Older Adults (SENIOR) project, also part of ON Foods, a third app was developed, namely Nutritional App-Senior (NutriA-S). This app was conceived because the staff at the Laboratory of Dietetics and Clinical Nutrition (LDNC) recognized the need to provide older adults with sarcopenia or probable sarcopenia with continuous nutritional support to strengthen and maintain healthy eating behaviors over time.

Sarcopenia is a progressive age-related muscle disease characterized by the loss of skeletal muscle mass, strength, and physical function. It reduces mobility, increases the risk of falls and fractures, and diminishes independent living. Nutrition plays a crucial role in countering sarcopenia because the aging body becomes less efficient at converting dietary nutrients into muscle tissue. Thus, older adults need a higher intake of high-quality protein to trigger muscle synthesis, a process that requires adequate literacy. In addition to nutrition, physical activity represents the other core dimension in counteracting sarcopenia. Physical exercise for older patients is carefully selected by the clinician and, once assigned, requires minimal daily decision-making from the patient [18-20]. Conversely, modifying eating habits is more demanding, since information received during clinical visits can be difficult to remember and implement in daily life without adequate reinforcement.

NutriA-S was designed as a simple-to-use tool that prioritizes nutrition education over self-monitoring of dietary intake, targeting issues especially relevant in later life and in frail populations, while providing nutrition-related management advice for clinical conditions affecting older adults. Unlike many existing nutrition apps, NutriA-S is a theory-informed, low-burden educational resource guided jointly by nutritional priorities and age-friendly usability principles. It aims to engage users through quizzes, informative content, and weekly challenges between scheduled visits, while also enabling remote monitoring of patient participation.

In this paper, we (1) present the rationale and design of NutriA-S, detailing its development and educational content, and (2) report the results of a pilot usability study conducted with age-matched healthy volunteers to assess user-perceived acceptability and collect actionable feedback for system refinement. This evaluation serves as a preparatory phase for launching a randomized controlled trial (RCT) in older adults with sarcopenia or probable sarcopenia.

## Methods

### Design of NutriA-S

The authors represent diverse research institutions and possess complementary expertise encompassing clinical nutrition, health behavior, information and communications technology (ICT), and data science. Adhering to established best practices for digital health development [21], this multidisciplinary team collaborated on the design of NutriA-S and analyzed the outcomes of the pilot usability study. Researchers at the Department of Electrical, Computer and Biomedical Engineering (Eng Dept) formalized the intervention’s underlying ICT infrastructure, designed the user experience logic, and structured the user interaction data collected for this pilot study, while staff at LDNC were responsible for generating educational content. The design process involved multiple iterative rounds of cross-disciplinary knowledge exchange. Thus, the clinical team was familiarized with smartphone app standards to ensure the interface was optimized for older adults. The technical team assisted in structuring the app’s interactive elements, ensuring that the clinical material was properly translated into a user-friendly digital format.

In the planned RCT, patients will receive standard in-person clinical care from LDNC staff for the first 6 months, after which NutriA-S will serve as a digital support tool to sustain engagement during the subsequent 6 months leading up to the final follow-up visit. To optimize this digital transition, the NutriA-S architecture was informed by the COM-B (capability, opportunity, motivation, and behavior) model and the Behavior Change Wheel (BCW) framework [22], which theorizes that behavior arises from the interaction between capability, opportunity, and motivation.

Specifically, NutriA-S targets psychological capability by delivering structured nutrition education through interactive quiz-based modules, explanatory information boxes, and immediate corrective feedback, thereby enhancing nutritional literacy and supporting informed dietary choices. Physical and social opportunities were addressed by ensuring high accessibility and minimizing technical barriers. This includes comprehensive technical onboarding at enrollment, storing login credentials locally after installation to minimize cognitive load, and planning a dedicated technical help desk throughout the study, accommodating caregiver support whenever necessary. Furthermore, NutriA-S interface elements were tailored to age-friendly usability standards, utilizing large text dimensions and high-contrast images while avoiding overly complex interaction modalities, based on the World Wide Web Consortium (W3C)’s mobile accessibility guidelines for aging users [23]. Finally, motivation was targeted through gamified quizzes, motivational feedback, and a progressive weekly content delivery schedule designed to sustain attention and encourage self-reflection. Weekly practical challenges were also introduced at the end of each module to maximize the real-world application of the acquired knowledge. Consequently, NutriA-S primarily operationalizes the education, persuasion, incentivization, environmental restructuring, and enablement intervention functions of the BCW, while other functions, such as modeling, restriction, coercion, and training, were excluded from this first app version to serve as potential avenues for future development.

On the implementation side, the design of NutriA-S was informed by existing literature addressing behavior change through mobile interventions. Ahmad et al [24] conducted a scoping review that analyzed barriers and motivators of mHealth interventions for older adults, identifying technological skills, beliefs about consequences, and memory, attention, and decision processes as the most critical domains to target for improving digital engagement in this population. Guided by these principles, NutriA-S was intentionally structured to minimize cognitive, physical, and operational burdens, aligning with additional evidence regarding mHealth apps [25-28].

The specific educational modules were chosen to reflect common nutritional inadequacies observed in older populations [19,20,29-33], focusing on insufficient consumption of plant-based foods (eg, vegetables and legumes) alongside inadequate intakes of energy, protein, dietary fiber, calcium, vitamin D, and water. Content was developed in accordance with current guidelines for the prevention and management of sarcopenia [18,34,35]. Furthermore, dedicated modules provided targeted dietary advice for managing clinical conditions prevalent in older adults, such as anemia, hypertension, diabetes, hypercholesterolemia, gastroesophageal reflux disease, and fall or fracture risks [36-40]. Collectively, these features were designed to ensure that the intervention was suitable for older adults with heterogeneous levels of digital literacy and for future deployment in patients with frailty.

### Nutritional Content of NutriA-S

The quiz format supported active learning, delivered immediate feedback to correct errors, and leveraged gamification to boost motivation. As shown in Table 1, a total of 48 interactive multiple-choice questions and 24 nutritional information items were created based on up-to-date literature references and classified into 5 thematic categories:

- Unlocking food facts for healthy habits: improve participants’ nutritional literacy by enhancing their understanding of macronutrients, micronutrients, and dietary guidelines, thereby supporting the adoption of evidence-based healthy eating behaviors [30,31].
- Staying safe with food—“tips for food safety”: raise awareness of critical food-safety principles, such as proper food handling, safe storage temperatures, and cross-contamination prevention, to reduce the risk of foodborne illnesses [31].
- Eating for the planet—“sustainability!”: educate participants on the environmental impacts of dietary patterns, such as greenhouse gas emissions, water footprints, and biodiversity loss, promoting sustainable food choices aligned with frameworks like the EAT-Lancet Commission recommendations [41-43].
- Preventing chronic noncommunicable diseases: increase knowledge of how nutrition modulates risk factors for chronic diseases, including cardiovascular diseases [44-46], type 2 diabetes [47], and obesity [48], highlighting evidence-based dietary patterns such as the Mediterranean and DASH (Dietary Approaches to Stop Hypertension) diets [31].
- Decoding food labels: build skills to interpret nutrition panels, ingredient lists, and health claims, enabling informed purchasing decisions in line with regulatory guidance (eg, the European Food Safety Authority) [49].

**Table 1.** Thematic categories and nutritional contents of Nutritional App-Senior (NutriA-S).

Main topics	Nutritional information items
Unlocking food facts for healthy habits	Healthy and balanced: the keys to eating well The rule of 5: your simplest path to lasting well-being! Health in a glass: what to drink and in what amounts Balancing taste and wellness: smart snack ideas Legumes and potatoes under the microscope Carbohydrates as a source of energy and well-being Fats: friends or foes? Protein power: a quick guide to understanding the best sources and benefits Protect your bones: how calcium and vitamin D work together? How does food fight anemia and keep your blood flowing smoothly? Fibers and wholegrain cereals: a winning combination for gut health Smart convenience: ready-to-eat products? A pinch of salt, a dash of sense
Staying safe with food: “tips for food safety”	When, where, and how: expiration dates and proper food storage Food safety in your kitchen
Eating for the plant: “sustainability!”	Blue gold: our most precious resource Greener shopping: bring sustainability to your grocery list! From field to table: good for your health, and even better for the planet
Preventing chronic noncommunicable diseases	Healthy habits, big wins: how lifestyle choices score against chronic diseases Beat the pressure: nutritional tips for tackling hypertension and diabetes Smart nutrition hacks to tackle hypercholesterolemia and gastroesophageal reflux
Decoding food labels	From A to Z: learn to read and understand food labels Low-content products: when and how to choose “low in…” options without falling for marketing traps Eating “Free From”: gluten and lactose explained

All questions and nutritional information items were grouped into 24 modules. Each module included 2 quizzes and 1 explanatory box that synthesized and briefly explained the quiz topics to encourage and guide the decision-making processes in patients. In the planned RCT, modules will be administered weekly over a 24-week period to avoid overwhelming patients while maintaining engagement and encouraging incremental dietary changes, as dictated by the memory and decision processes domain of the scoping review [24].

Each module was paired with a practical weekly challenge designed to help participants translate educational content into their daily routines. Examples include assembling a balanced plate using the Harvard Healthy Eating Plate framework (ie, a guide for healthy and balanced meals [50]), introducing a healthy between-meal snack, preparing traditional Tuscan soup, cooking nutrient-dense poultry dishes, experimenting with whole-grain bread or pasta varieties, and preparing baked fish. These challenges were intended to promote self-reflection and facilitate real-world knowledge application. Additionally, the platform delivered automated, tailored feedback based on quiz performance. This feedback was structured to acknowledge high baseline comprehension, highlight areas for further review, or identify concepts requiring fundamental improvement. Collectively, this architecture was designed to move beyond passive information delivery by synthesizing active corrective feedback, actionable goal setting, and repeated exposure to the educational material.

Finally, NutriA-S incorporated an ad hoc 10-item multiple-choice eating habits questionnaire to assess participants’ nutritional knowledge at baseline and study end point. This questionnaire was developed by the LDNC team based on the Italian dietary guidelines for healthy eating [31]. Aligned with the app’s educational objectives, the eating habits questionnaire covered core topics in nutrition, food safety, and sustainable eating [29,41]. Each correct response was awarded 1 point, yielding a total score that was categorized into 3 levels of nutritional knowledge: poor (0‐3 points), fair (4‐5 points), or good (6‐10 points). A brief format was selected, as shorter instruments have been shown to reduce respondent burden in older populations [51]. In the planned RCT, the comparison between initial and final scores will be used to assess knowledge retention over a 24-week period and, more broadly, to evaluate the app’s effectiveness in improving nutritional literacy.

### Technical Implementation

The Eng Dept staff addressed three main areas: (1) delivering questionnaires to assess users’ current conditions and habits; (2) administering quizzes and collecting answers; and (3) providing informational content so that users could explore the topics covered by the quizzes in greater depth, particularly when they obtained a low score.

Unlike our previous experiences in designing and implementing mobile apps for telemedicine systems [17,52,53], NutriA-S did not require complex interaction patterns among multiple parties for exchanging data. Therefore, rather than building the system from scratch, we structured it as an e-learning solution designed through close collaboration between nutrition experts and computer scientists, in line with other studies reported in the literature [54]. Specifically, we decided to leverage Moodle [55], the leading open-source e-learning platform widely used to deliver educational content in academic courses and assess learners’ proficiency through quizzes. Beyond its academic use, Moodle has been increasingly adopted in health care, although it has been more commonly used for training health care professionals [56] than for patient-oriented educational interventions, for which dedicated portals are usually preferred [57]. Nevertheless, we found that Moodle can also be highly suitable for patient-targeted apps by leveraging some of its specific features.

In addition to browser-based access via a PC, Moodle provides a dedicated mobile app for both Android and iOS. This approach was considered useful for simplifying access because our target population consists of older users, many of whom do not routinely use a PC. By contrast, most older adults now own a smartphone and have at least basic familiarity with mobile apps. Once the app is installed and configured with the proper credentials and the link to the associated Moodle server, users can carry out all interactions by simply tapping the app icon and navigating through the available links.

Figure 1 shows the NutriA-S deployment workflow and how the different actors interact with the app. Moodle was set up from scratch using the open-source codebase on a virtual machine hosted on Amazon Web Services. This choice was made to maximize service availability and to simplify system management, particularly if computational resources need to be scaled up during the future trial.

**Figure 1.**
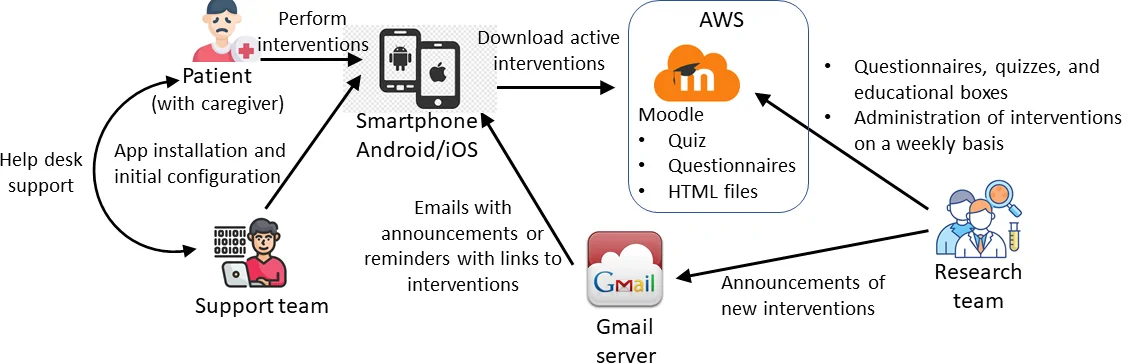
The overall architecture of Nutritional App-Senior (NutriA-S), illustrating its workflow and the different actors interacting with it. AWS: Amazon Web Services.

As displayed on the right side of Figure 1, the research team was responsible for creating NutriA-S content using the customary interface provided by Moodle for administrative purposes. To this end, we used Moodle’s quiz and questionnaire modules, together with simple files for the educational content that were rendered in HTML to embed images and include links to external reference websites. Among all the modules made available by Moodle, the quiz module was the most complex because it supported automatic grading by assigning scores to each possible answer. Thus, for each weekly quiz, scores were assigned based on answer correctness: 1 fully correct option yielded 100% of the score, 1 or 2 partially correct options yielded 50%, and 1 incorrect option yielded 0%. Scores were returned to the patient shortly after each weekly quiz as immediate feedback, along with a motivational message that directed the patient to the relevant nutritional information item to sustain engagement and support retention. Moreover, aggregating a patient’s score across all quiz categories at the end of the trial could help identify domains where further reinforcement might be needed.

The patient, shown on the left, interacts with the app either independently or with the support of a caregiver depending on their ability to use a smartphone. These skills are assessed prior to enrollment, together with verification that a suitable smartphone is available. During enrollment, a member of the support team assists with downloading and installing the Moodle mobile app, which is available through the major app stores (Google Play for Android and the Apple App Store for iOS). This step also includes configuring the app with the link to our Moodle installation and providing the patient with their credentials. Once set up, the credentials are stored by the app, so launching it subsequently requires only a single tap on the app icon. This was a key reason for preferring the mobile app, as it reduces the authentication burden typically associated with web-based applications, thereby addressing the need for minimal technological skills, as highlighted in the scoping review [24].

After a new patient is enrolled, the research team schedules the release of the educational modules and quizzes. This includes assigning an initial questionnaire and then introducing a weekly quiz paired with an educational box throughout the 24-week study period foreseen by the RCT. Once a new module is released, or if some degree of noncompliance is observed, reminder emails can be sent including a direct link that opens the app to the relevant module. If persistent noncompliance is observed (eg, the patient does not access NutriA-S for an extended period), the support team contacts the participant directly by phone. A dedicated help desk comprising both LDNC and Eng Dept research staff is also available throughout the study to remotely support the patient or caregiver.

Figure 2 presents screenshots illustrating NutriA-S’s visual appearance on a smartphone. Panel (A) shows the eating habits questionnaire administered at baseline, while panel (B) depicts an example quiz question screen. Finally, panel (C) displays a nutritional information item. NutriA-S was developed in Italian to match users’ native language. However, selected sections were translated into English for research and documentation purposes, including the content displayed in Figure 2.

**Figure 2.**
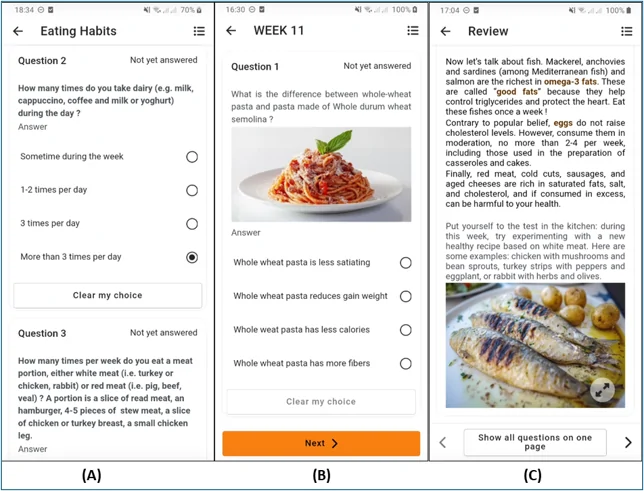
Sample of 3 screenshots taken on a smartphone running Nutritional App-Senior (NutriA-S).

### Usability Study

After development was completed, a 4-week pilot study was conducted to assess the usability of NutriA-S. Participants were recruited through flyers posted in October 2025 in 2 outpatient settings: the LDNC at the University of Pavia (Pavia, Italy) and the outpatient clinic of Endocrinology and Metabolism at the Istituti Clinici Scientifici Maugeri (Pavia, Italy). Healthy volunteers who met the eligibility criteria were invited to participate. Recruitment involved relatives and acquaintances of patients attending the 2 outpatient clinics. Eligibility criteria included self-reported good general health (ie, no major chronic or acute medical conditions affecting nutritional status at the time of enrollment) and age between 65 and 85 years. Given the exploratory nature of this pilot usability study, participants were restricted to those aged 65 to 85 years to reduce heterogeneity related to very advanced age, including sensory, functional, and cognitive factors that could independently influence usability outcomes. This age range was considered appropriate for an initial usability evaluation before extending testing to the oldest-old population in future studies.

The research team provided individualized technical support to assist each participant with downloading and navigating the platform. After recruitment, the study was conducted entirely remotely. Participants used the app from their homes and were monitored via established telemedicine procedures. Additionally, a dedicated LDNC team member was assigned to each participant as the primary point of contact. Detailed flyers, containing step-by-step procedures, help desk information, and direct contact details were distributed to ensure that support was readily accessible whenever needed. All participants were informed of the study aims and procedures prior to enrollment.

The usability study followed the same deployment workflow planned for the subsequent clinical study, but on a shorter time scale, as participants had access to the app for only 4 weeks. Before configuring the app, a member of the LDNC team collected baseline demographic variables, such as age, sex, educational level, and place of residence. Although some of these variables were not strictly necessary for the usability assessment, they were collected because they will be required in the future RCT and allowed potential issues in the data-collection process to be identified. When a caregiver was present, data on the caregiver’s age, educational level, and relationship to the participant (family member or professional) were also recorded. If the caregiver was the primary user of the app, this was noted. Digital literacy of the participant and/or caregiver (if present) was assessed using the following 5-level scale: (1) very poor: does not know what an app is and uses the smartphone only for calls; (2) poor: rarely uses apps and has difficulty navigating between screens or interpreting messages; (3) fair: understands what apps are and uses them at a basic level, with some difficulties in configuration (eg, logging back into Moodle after session expiration); (4) good: regularly uses apps and may occasionally overlook details, especially when facing a new app; (5) very good: fully comfortable installing, configuring, and using apps on their smartphone.

During the first week, participants were asked to complete the eating habits questionnaire delivered through NutriA-S. The results were used to describe baseline dietary habits and skills in a population comparable to the future RCT population. After the first week, all 24 quizzes were unlocked at once, along with the associated nutritional information items, rather than releasing 1 quiz per week as planned for the RCT. This choice was made to allow users to freely explore the entire app content within a shorter period. In fact, given its usability-oriented focus, this pilot study was not intended to demonstrate retention or definitive dietary behavior change; rather, it aimed to determine whether older adults could effectively access, navigate, and engage with the app, thereby guiding system refinements prior to the planned RCT.

The choice of the population for this pilot study involved convenience and self-selected sampling, which may introduce biases related to participant motivation, digital literacy, cognitive ability, health status, and need for caregiver support. Future studies should evaluate usability among adults aged over 85 years, who represent an important subgroup of the target population and may have specific accessibility needs.

### Ethical Considerations

The reference ethics committee for the study was the Comitato Etico Territoriale Lombardia 6 (CET L6) at Fondazione Policlinico San Matteo (Pavia, Italy). The pilot usability study was embedded within the SENIOR study, which was approved by the Ethics Committee of Istituti Clinici Scientifici Maugeri (2782 CE, protocol 0043995/24) and by the Ethics Committee of San Gerardo Hospital, University of Milan-Bicocca (code: 4881_11.09.2024_M). The amendment for the pilot usability study was approved under protocol 0068982/25.

All eligible participants read and signed an informed consent form before taking part in the study. When caregivers were involved, they also provided written informed consent. To protect participants’ privacy and confidentiality, all data were anonymized using a study-specific code in the format t00x. No participant compensation was provided.

### Usability Questionnaires and Qualitative Feedback

At the end of the 4-week study period, a member of the LDNC team interviewed each participant, administering the Italian versions of the System Usability Scale (SUS) [58] and the User version of the Mobile Application Rating Scale (uMARS) [59] questionnaires. The questionnaires were administered after providing a clear explanation of the instructions, as a means of assessing the usability of the app. Immediately after their completion, the interviewer invited participants to provide additional comments, which were recorded verbatim in written form. This allowed users to self-report perceived strengths or shortcomings of NutriA-S to inform future system improvements.

The SUS is a validated, simple 10-item questionnaire designed to assess the usability of a generic technical device or equipment, not necessarily related to information technology. Each item allows 5 response options based on a Likert scale, ranging from 1 to 5. Its distinguishing feature is that for the odd items (ie, 1, 3, 5, 7, and 9), a response of 1 means “strongly disagree,” while a response of 5 means “strongly agree.” For the even items (ie, 2, 4, 6, 8, and 10), the meaning is reversed, with 1 meaning “strongly agree” and 5 meaning “strongly disagree.” This quirk is designed to prevent bias arising from users answering 1 or 5 to all items.

uMARS is a validated questionnaire specifically designed to capture the usability of mobile apps by end users. uMARS is structured into 4 main sections (ie, engagement, functionality, aesthetics, and information), each including 3 to 5 items and 2 additional sections (ie, subjective quality and perceived impact) with 4 to 6 items.

### Statistical Analyses

Questionnaire responses were converted to a 0 to 4 scale, aligning item polarity and ensuring that higher values consistently indicate better usability. For positively worded items, the score was calculated as the raw response minus 1. Conversely, for negatively worded items, the score was calculated as 5 minus the raw response. The total SUS score was calculated by summing the scores of all 10 items and multiplying the result by 2.5, yielding a final score ranging from 0 to 100. Overall, SUS scores were categorized using conventional thresholds as acceptable (≥68), marginal (50-67), or not acceptable (<50) [60,61]. For uMARS, we analyzed both the total score and the scores for each subscale (engagement, functionality, aesthetics, information and the additional dimensions, subjective quality, and perceived impact) score.

We explored the correlation between SUS and uMARS scores using Spearman *r*. Additionally, we tested differences in overall usability scores (SUS and uMARS) and in each uMARS subscale across groups defined by digital literacy, dietary habits, education, caregiver presence, and gender. Normality was assessed with the Shapiro-Wilk test. Between-group comparisons were performed using *t* tests (2 levels) or ANOVA (more than 2 levels) for normally distributed variables, and Wilcoxon rank-sum or Kruskal-Wallis tests otherwise, with Benjamini-Hochberg correction for multiple comparisons. All analyses were conducted in R software (v4.3.3; R Foundation for Statistical Computing).

## Results

### Participant Characteristics

Table 2 summarizes baseline demographics of user participants. In total, 35 volunteers (n=17, 48.57% were women) completed the usability study. The median age was 69.03 (IQR 66.33-73.92) years. Regarding education, over 71.43% (n=25) had completed high school, and 4 out of 35 had also attended university. Concerning ICT literacy, most participants had at least basic app skills, such as browsing and logging in independently, and 7 out of 35 reported optimal proficiency. Nine (25.71%) participants needed caregiver support to use the app (5 were assisted by a son, 2 by a nephew, and 2 by a partner). The median caregiver age was 39.5 (IQR 27.25-57.50) years.

**Table 2.** Participant demographic, educational, digital literacy, and dietary habits characteristics.

Characteristics	Values (N=35)
Gender, n (%)	
Women	17 (48.57)
Men	18 (51.43)
Age (y), median (IQR)	69.03 (7.59)
Caregiver availability, n (%)	9 (25.71)
Education, n (%)	
Elementary school	2 (5.71)
Middle school	8 (22.86)
High school	21 (60)
University or above	4 (11.43)
Digital literacy, n (%)	
Very poor	0 (0)
Poor	2 (5.71)
Fair	17 (48.57)
Good	9 (25.71)
Very good	7 (20)
Eating habits, n (%)	
Poor	11 (31.43)
Fair	12 (34.29)
Good	12 (34.29)

### SUS Rating

Overall, NutriA-S had a median SUS total score of 80 out of 100 (IQR 55-92.50), indicating good perceived usability. More than half of the participants rated the app as acceptable, and 12 of 35 (34.29%) rated it at least marginal (with 3 users scoring 67.5, just below the 70-point threshold for acceptable), while 5 of 35 (14.29%) participants provided a total score below 50 (not acceptable).

Table 3 reports an exploration of item-level distributions after aligning item polarity. Question 3 (ease of use), question 6 (perceived inconsistency), question 8 (cumbersomeness), and question 10 (amount of learning required) show the highest scores on the (0‐4) scale. The only item with a lower central tendency, although still in the middle, compared to the other statements was question 1 (intention to use the system frequently), with a median of 2 on the (0‐4) scale.

**Table 3.** Summary statistics of System Usability Scale (SUS) single-item scores after polarity alignment to a common 0 to 4 scale (higher is better).

SUS item	Score, median (IQR)
Q1^a^ (frequency)	2 (2-3)
Q2 (complexity)	3 (2-4)
Q3 (ease of use)	4 (2-4)
Q4 (need for support)	3 (2-4)
Q5 (integration)	3 (2-4)
Q6 (inconsistency)	4 (3-4)
Q7 (learnability)	3 (2-4)
Q8 (cumbersomeness)	4 (2-4)
Q9 (confidence)	3 (2-4)
Q10 (learning curve)	4 (2-4)

a Q: question.

### uMARS Rating

Table 4 summarizes uMARS subscale scores across the 4 core dimensions (ie, engagement, functionality, aesthetics, and information) used to compute the total score and also reports 2 additional sections, namely subjective quality and perceived impact. The overall median uMARS score was 4.08 (IQR 3.68-4.30). Functionality and information showed the highest median score of 4.5 (IQR 4-4.50 and IQR 4.13-4.75, respectively), followed by aesthetics (median=4.0, IQR 3.33-4.17). No statistically significant differences were observed across digital literacy, food habits, education, caregiver presence, or gender.

**Table 4.** Summary statistics of User Version of the Mobile Application Rating Scale (uMARS) subscale scores.

uMARS subscale	Score, median (IQR)
Engagement	3.60 (3‐3.90)
Functionality	4.50 (4‐4.50)
Aesthetics	4 (3.33‐4.17)
Information	4.50 (4.13‐4.75)
Subjective quality	3.25 (2.75‐3.50)
Perceived impact	4 (3.33‐4.75)

Table 5 presents summary statistics for each individual uMARS item, grouped by subscale. The highest item scores (median 5, IQR 4-5) were observed in functionality and information, specifically for items on performance (median 5, IQR 4-5), amount of information (median 5, IQR 4-5), and source credibility (median 5, IQR 4.5-5), as well as in perceived impact for the awareness item (median 5, IQR 4-5). Subjective quality had the lowest subscale median (3.25, IQR 2.75-3.50), but only 1 item (ie, payment) showed a median less than 3 (median 2, IQR 1-2.50). Moreover, although the customization item within the engagement subscale had a median 3 (IQR 1-3), it showed a wide dispersion, with several low ratings.

**Table 5.** Summary statistics of User Version of the Mobile Application Rating Scale (uMARS) single-item scores.

uMARS subscale and item	Score, median (IQR)
Engagement	
Entertainment	4 (3-4)
Interest	4 (4-5)
Customization	3 (1-3)
Interactivity	3 (3-3)
Target group	4 (3-5)
Functionality	
Performance	5 (4-5)
Ease of use	4 (4-5)
Navigation	4 (4-4)
Gestural design	4 (4-5)
Aesthetics	
Layout	4 (3.50‐4)
Graphics	4 (3-4)
Visual appeal	4 (3-4)
Information	
Quality	4 (4-4)
Quantity	5 (4-5)
Visual	4 (4-5)
Credibility of source	5 (4.50‐5)
Subjective quality	
Recommendation to others	4 (3-5)
Use and relevance	3 (2-4)
Payment	2 (1‐2.50)
Overall rating	4 (3-4)
Perceived impact	
Awareness	5 (4-5)
Knowledge	4 (3.50‐5)
Attitudes	4 (3-4)
Intention to change	4 (3-5)
Help seeking	4 (3-5)
Behavior change	4 (3-5)

Overall, uMARS scores were positively correlated with SUS scores (Spearman *r*=0.57, *P*<.001), indicating that participants who perceived the app as more usable on the SUS also tended to rate its overall quality more positively on the uMARS.

### Qualitative Feedback

After completing the SUS and uMARS questionnaires, participants were invited to provide additional suggestions, but only a few participants (8 out of 35) took advantage of this opportunity. Some users provided general comments about the overall study rather than feedback specifically related to NutriA-S, while a subset of comments directly addressed the perceived usefulness of the app, offering valuable insights that can inform future improvements. No formal coding or thematic analysis was conducted, given the very limited number of feedback comments.

Among these responses, there was a notable interest in advanced app features and personalization. Two users expressed a desire for customizable content, specifically requesting tailored recipes to promote the intake of less frequently consumed foods. Nevertheless, another participant perceived such personalization as potentially overcomplicated and unnecessary due to their unfamiliarity with similar apps. Finally, user engagement levels varied notably. While 1 user actively sought more functionality, such as the ability to review completed quizzes later and access more external links to further explore dietary topics, another admitted to interacting with the app only for the minimum duration required to complete the quizzes.

A few participants also commented on the usefulness of the app for activities of daily living, specifically in the context of grocery shopping. One participant reported finding the sections on reading food labels and on low-content products helpful while shopping at the supermarket. Another noted that using the app in the supermarket allowed them to draw on ideas from all modules about what to buy in order to eat healthily.

## Discussion

### Principal Findings

In this paper, we detailed the design and implementation framework for NutriA-S, a dedicated mHealth app designed to optimize nutritional education for older adults with sarcopenia or probable sarcopenia, and evaluated its usability among age-matched healthy volunteers prior to formal clinical validation.

Overall, NutriA-S showed encouraging usability results. The median SUS score reached 80 out of 100 (IQR 55-92.50), above the conventional acceptability threshold of 68 [58], indicating high usability. The median uMARS score was 4.083 out of 5 (IQR 3.68-4.3), with the functionality and information domains emerging as the highest-rated dimensions. These findings suggest that older adults were able to effectively access, navigate, and interact with the app and that they perceived its educational content and interface as credible and intuitive. This is largely attributable to our efforts to minimize cognitive, operational, and visual burdens by using a streamlined layout, simplified language, and age-friendly design principles [62]. Conversely, lower scores observed for frequency of use, customizability, and willingness to pay indicate areas that should be refined before testing NutriA-S in older adults with sarcopenia or probable sarcopenia.

While several mHealth apps have been developed in recent years to support nutrition, the majority focus on weight management and chronic metabolic conditions such as type 2 diabetes [63]. Most solutions offer self-monitoring and coaching features, whereas structured educational content remains sparse. In addition, it is reported that interactive feedback, gamification, and questionnaires are implemented least frequently, and very few solutions have been designed or evaluated exclusively for older populations [63].

The digital nutrition landscape for healthy older adults and frail cohorts remains highly heterogeneous across aims, theoretical foundations, and evaluation methodologies. Among apps evaluated in healthy or independently living older adults, Appetitus and NuMob-e-App (Carl von Ossietzky University Oldenburg) provide valuable benchmarks [64,65]. Appetitus was introduced within a broader nutritional educational intervention for healthy community-dwelling older adults [64]. Although the intervention was feasible and increased nutritional awareness, most participants did not use the app afterward because longitudinal dietary tracking was deemed time-consuming and not useful in everyday life in the absence of an immediate health need. Interestingly, a user noted Appetitus’s potential utility during postsurgical recovery, suggesting that its perceived value may increase when nutritional monitoring is linked to a concrete clinical outcome. Similarly, NuMob-e-App was introduced as a tablet-based dietary record tool for adults aged 70 years or older featuring age-adapted interfaces [65]. While the app demonstrated relative validity across several dietary variables, systematic underestimation compared with 24-hour recalls indicated a need for further refinement. Overall, these findings support the potential of age-adapted digital tools in healthy older populations, but also highlight that perceived usefulness, low registration burden, and personalized feedback are critical determinants of long-term compliance.

When considering digital interventions designed specifically for frail, malnourished, or sarcopenic older populations, additional structural and methodological challenges emerge [66-69]. The app Olitor (Oleg Zaslavsky) was evaluated in a pilot RCT, suggesting potential improvements in Mediterranean diet adherence among frail geriatric patients [66]. Food’n’Go was designed to support appetite and food intake in hospitalized older patients at risk of malnutrition. Technical immaturity of the app, including unplanned device restarts and login problems, undermined independent home use after discharge [67-69]. Conversely, KOKU-Nut (University of Manchester in collaboration with Reason Digital) represented a participatory approach, involving community-dwelling older adults in the development of a game-based nutrition tool to prevent nutritional risk. The app reported promising pilot usability findings, although comprehensive feasibility trial outcomes have not yet been fully published [70,71]. Although findings in healthy older adults were not encouraging, results from Appetitus use appeared more favorable among older adults at risk of malnutrition, among whom the app seemed promising in promoting awareness of dietary intake. Nevertheless, Appetitus remained primarily oriented toward dietary self-monitoring rather than formal nutrition education, and usability was not assessed through standardized instruments [64].

In contrast to these app paradigms, NutriA-S was designed primarily as a structured educational and motivational mHealth app tailored for older adults, rather than a food diary or dietary intake monitoring system. Its quiz-based format, weekly educational modules, active goal-setting challenges, and immediate feedback were intended to incentivize participation, promote active learning, and support self-reflection regarding dietary choices. This educational focus is particularly relevant for older adults with sarcopenia or probable sarcopenia. For this frail population, clinical guidelines recommend not only monitoring intake but also actively improving comprehension of energy density, protein requirements, hydration, micronutrient adequacy, food safety, and diet-related management of common chronic conditions [18,34,35].

Regarding the usability outcomes, the median SUS score of 80 (IQR 55-92.5) observed for NutriA-S is aligned with the upper range of usability reported for comparable digital nutrition tools [72-77], despite the well-documented accessibility barriers associated with older age and heterogeneous levels of digital literacy. For instance, tablet-based e-coach systems developed for geriatric rehabilitation settings have shown progressive improvements in usability across iterative design cycles, with SUS scores shifting from acceptable baselines to good or excellent ranges following user-informed modifications [72,74]. Similarly, a pilot digital assistive system combining a food diary with mobility monitoring achieved a mean SUS score close to 80 among adults aged 70 years and older [73]. Other dietary tools, including a voice-supported mobile food reporting app evaluated in an RCT [76] and the graphical food-frequency app eNutri [77], further support the feasibility of achieving high usability scores in older adults. Notably, participants gave relatively low ratings only to the specific item regarding projected frequency of use. This trend may suggest that as users acquire nutritional literacy and healthier eating habits, they view the app as less of a daily necessity. Alternatively, a low intended frequency of use could indicate that the platform requires further refinement to enhance independent user engagement.

A significant methodological contribution of the present study is the combined use of SUS and uMARS. While SUS allows comparison with a broader body of usability studies in older adults [78], uMARS provides a multidimensional assessment across engagement, functionality, aesthetics, information quality, subjective quality, and perceived impact [59]. In our sample, a moderate, statistically significant, positive correlation was observed between the 2 scales (*r*=0.57, *P*<.001). This indicates that participants who perceived the app as more usable on the SUS also tended to rate its overall quality more positively on the uMARS, suggesting that the 2 questionnaires assess related yet distinct constructs. Using both scales during this pilot usability study allowed the research team to gather highly granular data across specific software quality dimensions that are inherently captured by the uMARS but are absent from the SUS. Conversely, only the SUS will be administered during the subsequent RCT. Given that the SUS is significantly shorter and cognitively less demanding, its exclusive use will minimize respondent burden while still providing a reliable, standardized, and validated metric of overall system usability in older adults with sarcopenia or probable sarcopenia.

The high ratings observed in this study for the functionality and information uMARS subscales suggest that older participants perceived NutriA-S as both technically robust and highly credible in its educational content. Conversely, the lower scores recorded for the subjective quality subscale highlight specific areas for future development. Subsequent iterations of NutriA-S should incorporate tailored personalization features, such as optional automatic reminders, customized recipes, or links to additional nutritional resources, while carefully avoiding operational complexity for users with limited digital literacy. Although some of the weekly challenges concerned dish composition or culinary suggestions, only basic principles were provided, rather than complete, precisely measured recipes. Furthermore, some of the lower ratings recorded within the subjective quality subscale of uMARS can be contextualized. For instance, the low uMARS score for the willingness-to-pay item may reflect expectations shaped by the Italian health care system, which provides comprehensive coverage of medical expenses based on age and income. Consequently, participants may implicitly expect digital health interventions like NutriA-S to be free of charge. Nevertheless, this finding should not be dismissed, as a limited willingness to pay could also suggest that nutrition is still perceived as a secondary component of wellness rather than a primary pillar of healthy aging.

### Study Limitations

This study has several limitations that should be acknowledged. First, as a pilot usability study, this trial was characterized by a relatively small sample size and was neither designed nor statistically powered to evaluate clinical outcomes or long-term dietary behavior change. Second, the evaluation was conducted with healthy older volunteers, recruited via a convenience and self-selected sampling approach. This strategy introduced a selection bias, as our participants may differ substantially from the target end users, namely older adults with sarcopenia or probable sarcopenia, across critical dimensions such as health status, baseline motivation, digital literacy, cognitive ability, and the need for caregiver support. Therefore, usability, engagement, and retention may differ when NutriA-S is deployed in patients with frailty, malnutrition, or sarcopenia. Third, the usability study lasted only 4 weeks, whereas the planned intervention in the RCT will span 24 weeks. Consequently, this brief evaluative window prevents any definitive conclusions regarding long-term therapeutic adherence, sustained digital engagement, or longitudinal knowledge retention over extended periods. Fourth, the study did not include a comparator group receiving conventional paper-based nutrition education, leaving the exact relative advantage of the interactive digital format over traditional educational materials unquantified at this stage.

### Conclusions

This pilot study indicates that NutriA-S is a highly usable, intuitive, and credible digital intervention for older adults. More broadly, these results suggest that older populations are not fundamentally averse to digital health technologies; rather, their engagement depends on intuitive and low-burden design paradigms that offer immediate practical utility.

To secure long-term adherence during the planned RCT, the upcoming version of NutriA-S will explore the inclusion of low-complexity gamified elements, such as digital badges and progression milestones, which have proven effective and highly appreciated among older demographics [79]. Furthermore, in response to the qualitative feedback collected during this pilot study, features such as integrated links to tailored recipe videos will be explored in addition to the current links to authoritative scientific sources, satisfying the user demand for deeper practical resources without complicating the primary interface.

A future version of NutriA-S will also explore the introduction of selected personalization features, such as reminders and customizable layouts. Given that these options were initially excluded to safeguard the target population from cognitive fatigue, their future integration will be carefully designed to prevent the operational complexity that could compromise usability for users with limited digital literacy. Finally, given the theory-informed nature of the app, future development could incorporate additional functions addressing other components of the BCW, such as modeling, restriction, coercion, and training, to further strengthen the intervention’s behavioral impact while strictly preserving its streamlined and accessible design.

## Abbreviations

BCW: Behavior Change Wheel
CET L6: Comitato Etico Territoriale Lombardia 6
COM-B: capability, opportunity, motivation, and behavior
DASH: Dietary Approaches to Stop Hypertension
Eng Dept: Department of Electrical, Computer and Biomedical Engineering
IBD: inflammatory bowel disease
ICT: information communication technology
LDNC: Laboratory of Dietetics and Clinical Nutrition
mHealth: mobile health
NutriA: Nutritional App
NutriA-S: Nutritional App-Senior
RCT: randomized controlled trial
SENIOR: Sustainable Eating Patterns to Limit Malnutrition in Older Adults
SUS: System Usability Scale
uMARS: User Version of the Mobile Application Rating Scale
W3C: World Wide Web Consortium

## References

[1] Hamine S, Gerth-Guyette E, Faulx D, Green BB, Ginsburg AS (2015). Impact of mHealth chronic disease management on treatment adherence and patient outcomes: a systematic review. J Med Internet Res.

[2] Lanzola G, Capozzi D, D’Annunzio G, Ferrari P, Bellazzi R, Larizza C (2007). Going mobile with a multiaccess service for the management of diabetic patients. J Diabetes Sci Technol.

[3] Hoque R, Sorwar G (2017). Understanding factors influencing the adoption of mHealth by the elderly: an extension of the UTAUT model. Int J Med Inform.

[4] Wang Y, Yan P, You S (2025). mHealth-based educational needs in older adults with declines in intrinsic capacity: a qualitative research study. Geriatr Nurs.

[5] Schumer H, Amadi C, Joshi A (2018). Evaluating the dietary and nutritional apps in the Google Play Store. Healthc Inform Res.

[6] Mejia C, Libby BA, Bracken ML (2021). Interest in digital dietary support among adults with kidney failure receiving hemodialysis. J Ren Nutr.

[7] Karduck J, Chapman-Novakofski K (2018). Results of the Clinician Apps Survey, how clinicians working with patients with diabetes and obesity use mobile health apps. J Nutr Educ Behav.

[8] Chen J, Lieffers J, Bauman A, Hanning R, Allman-Farinelli M (2017). The use of smartphone health apps and other mobile health (mHealth) technologies in dietetic practice: a three country study. J Hum Nutr Diet.

[9] Maunder K, Walton K, Williams P, Ferguson M, Beck E (2018). eHealth readiness of dietitians. J Hum Nutr Diet.

[10] Dusi R, Trombini RRSL, Pereira ALM (2024). Construction and content validation of mobile devices’ application messages about food and nutrition for DM2 older adults. Nutrients.

[11] Ng KLS, Munisamy M, Lim JBY, Alshagga M (2025). The effect of nutritional mobile apps on populations with cancer: systematic review. JMIR Cancer.

[12] Kalimullah K, Sushmitha D (2017). Influence of design elements in mobile applications on user experience of elderly people. Procedia Comput Sci.

[13] Wildenbos GA, Peute L, Jaspers M (2018). Aging barriers influencing mobile health usability for older adults: a literature based framework (MOLD-US). Int J Med Inform.

[14] Vietzke J, Schenk L, Baer NR (2023). Middle-aged and older adults’ acceptance of mobile nutrition and fitness tools: a qualitative typology. Digit Health.

[15] OnFoods.

[16] Petrini G, Pala D, Lenti MV (2024). NutriA: a responsive web app to monitor nutrition and clinical outcomes in inflammatory bowel disease patients. 2024 IEEE Int Conf Bioinformatics Biomed (BIBM).

[17] Larizza C, Bosoni P, Quaglini S (2023). V-care: an application to support lifestyle improvement in children with obesity. Int J Med Inform.

[18] Cruz-Jentoft AJ, Sayer AA (2019). Sarcopenia. Lancet.

[19] Volkert D, Beck AM, Cederholm T (2019). ESPEN guideline on clinical nutrition and hydration in geriatrics. Clin Nutr.

[20] Deutz NEP, Bauer JM, Barazzoni R (2014). Protein intake and exercise for optimal muscle function with aging: recommendations from the ESPEN expert group. Clin Nutr.

[21] Toh SH, Davis C, Bte Khaider K, Ong ZQ, Lim EJK, Chew CSE (2025). Codevelopment of an mHealth app with health care providers, digital health experts, community partners, and families for childhood obesity management: protocol for a co-design process. JMIR Res Protoc.

[22] Michie S, van Stralen MM, West R (2011). The behaviour change wheel: a new method for characterising and designing behaviour change interventions. Implement Sci.

[23] Older users and web accessibility: meeting the needs of ageing web users. W3C Web Accessibility Initiative (WAI).

[24] Ahmad NA, Mat Ludin AF, Shahar S, Mohd Noah SA, Mohd Tohit N (2020). Willingness, perceived barriers and motivators in adopting mobile applications for health-related interventions among older adults: a scoping review protocol. BMJ Open.

[25] Gomez-Hernandez M, Ferre X, Moral C, Villalba-Mora E (2023). Design guidelines of mobile apps for older adults: systematic review and thematic analysis. JMIR mHealth uHealth.

[26] Li C, Neugroschl J, Zhu CW (2021). Design considerations for mobile health applications targeting older adults. J Alzheimers Dis.

[27] Amouzadeh E, Dianat I, Faradmal J, Babamiri M (2025). Optimizing mobile app design for older adults: systematic review of age-friendly design. Aging Clin Exp Res.

[28] Sobrinho ACDS, Gomes GAO, Bueno Júnior CR (2024). Developing a multiprofessional mobile app to enhance health habits in older adults: user-centered approach. JMIR Form Res.

[29] Mistura L, Donne CL, D’Addezio L (2025). The Italian IV SCAI dietary survey: main results on food consumption. Nutr Metab Cardiovasc Dis.

[30] LARN Tables 2014 [Article in Italian].

[31] (2029). Linee guida per una sana alimentazione: revisione 2018 (dietary guidelines for healthy eating: 2018 revision) [Report in Italian]. https://www.crea.gov.it/web/alimenti-e-nutrizione/-/linee-guida-per-una-sana-alimentazione-2018.

[32] Finta H, Avram C, Buicu CF, Ceana DE, Moldovan I, Ruta F (2026). Micronutrient status, health implications, and assessment approaches in older adults: a narrative review of recent studies. Life (Basel).

[33] Veronese N, Gianfredi V, Volpe M (2026). 2025 national guidelines on the Mediterranean diet: executive summary of a joint report by Italian scientific societies and the National Institute of Health Task Force on Clinical Practice Guidelines. Nutr Rev.

[34] Sayer AA, Cooper R, Arai H (2024). Sarcopenia. Nat Rev Dis Primers.

[35] Calvani R, Picca A, Coelho-Júnior HJ, Tosato M, Marzetti E, Landi F (2023). Diet for the prevention and management of sarcopenia. Metabolism.

[36] (2021). Decade of healthy ageing: baseline report. https://www.who.int/publications/i/item/9789240017900.

[37] Kernick D, Chew-Graham CA, O’Flynn N (2017). Clinical assessment and management of multimorbidity: NICE guideline. Br J Gen Pract.

[38] American Diabetes Association Professional Practice Committee for Diabetes* (2026). 13. Older Adults: Standards of Care in Diabetes-2026. Diabetes Care.

[39] Whelton PK, Carey RM, Aronow WS (2018). 2017 ACC/AHA/AAPA/ABC/ACPM/AGS/APhA/ASH/ASPC/NMA/PCNA guideline for the prevention, detection, evaluation, and management of high blood pressure in adults: a report of the American College of Cardiology/American Heart Association Task Force on Clinical Practice Guidelines. Hypertension.

[40] Boyd C, Smith CD, Masoudi FA (2019). Decision making for older adults with multiple chronic conditions: executive summary for the American Geriatrics Society guiding principles on the care of older adults with multimorbidity. J Am Geriatr Soc.

[41] Willett W, Rockström J, Loken B (2019). Food in the Anthropocene: the EAT-Lancet Commission on healthy diets from sustainable food systems. Lancet.

[42] The 17 goals. United Nations—Sustainable Development.

[43] World Commission on Environment and Development (1987). Our Common Future.

[44] McEvoy JW, McCarthy CP, Bruno RM (2024). 2024 ESC Guidelines for the management of elevated blood pressure and hypertension. Eur Heart J.

[45] Marx N, Federici M, Schütt K (2023). 2023 ESC Guidelines for the management of cardiovascular disease in patients with diabetes. Eur Heart J.

[46] Mach F, Baigent C, Catapano AL (2020). 2019 ESC/EAS Guidelines for the management of dyslipidaemias: lipid modification to reduce cardiovascular risk. Eur Heart J.

[47] Davies MJ, D’Alessio DA, Fradkin J (2018). Management of hyperglycemia in type 2 diabetes, 2018. A consensus report by the American Diabetes Association (ADA) and the European Association for the Study of Diabetes (EASD). Diabetes Care.

[48] Rubino F, Cummings DE, Eckel RH (2025). Definition and diagnostic criteria of clinical obesity. Lancet Diabetes Endocrinol.

[49] European food safety authority (EFSA). Food date labelling.

[50] Healthy eating plate. The Nutrition Source.

[51] Rolstad S, Adler J, Rydén A (2011). Response burden and questionnaire length: is shorter better? A review and meta-analysis. Value Health.

[52] Capozzi D, Lanzola G (2013). A generic telemedicine infrastructure for monitoring an artificial pancreas trial. Comput Methods Programs Biomed.

[53] Zini EM, Lanzola G, Quaglini S, Bossi P, Licitra L, Resteghini C (2019). A pilot study of a smartphone-based monitoring intervention on head and neck cancer patients undergoing concurrent chemo-radiotherapy. Int J Med Inform.

[54] Stotz S, Lee JS (2018). Development of an online smartphone-based eLearning nutrition education program for low-income individuals. J Nutr Educ Behav.

[55] Moodle.

[56] Jeong S, Hwang H (2023). Do we need Moodle in medical education? A review of its impact and utility. Kosin Med J.

[57] Serrato CA, Retecki S, Schmidt DE (2007). MyChart—A new mode of care delivery: 2005 Personal Health Link research report. Perm J.

[58] Brooke J, Jordan PW, Thomas B, McClelland IL, Weerdmeester B (1996). Usability Evaluation In Industry.

[59] Stoyanov SR, Hides L, Kavanagh DJ, Wilson H (2016). Development and validation of the User Version of the Mobile Application Rating Scale (uMARS). JMIR mHealth uHealth.

[60] Lewis JR (2018). The system usability scale: past, present, and future. Int J Hum-Comput Interact.

[61] Bangor A, Kortum PT, Miller JT (2008). An empirical evaluation of the system usability scale. Int J Hum-Comput Interact.

[62] Harte R, Glynn L, Rodríguez-Molinero A (2017). A human-centered design methodology to enhance the usability, human factors, and user experience of connected health systems: a three-phase methodology. JMIR Hum Factors.

[63] Pala D, Petrini G, Bosoni P, Larizza C, Quaglini S, Lanzola G (2024). Smartphone applications for nutrition support: a systematic review of the target outcomes and main functionalities. Int J Med Inform.

[64] Aakre JA, Holteng LBA, Testad I (2023). Healthy community-dwelling older adults’ experience with a nutritional educational intervention: a feasibility study. Gerontechnology.

[65] Hinrichsen J, Quinten V, Diekmann R (2025). Validation and evaluation of a tablet-based dietary record app for adults aged 70 and above. PLoS One.

[66] Su Y, Wu KC, Chien SY, Naik A, Zaslavsky O (2023). A mobile intervention designed specifically for older adults with frailty to support healthy eating: pilot randomized controlled trial. JMIR Form Res.

[67] Terp R, Kayser L, Lindhardt T (2021). Older patients’ competence, preferences, and attitudes toward digital technology use: explorative study. JMIR Hum Factors.

[68] Terp R, Lindhardt T, Kayser L (2022). Theory-driven development of an educative nutritional intervention (ENI) supporting older hospital patients to eat sufficiently, assisted by an eHealth solution: an intervention mapping approach. BMC Health Serv Res.

[69] Terp R, Kayser L, Lindhardt T (2024). An educative nutritional intervention supporting older hospital patients to eat sufficiently using eHealth: a mixed methods feasibility and pilot study. BMC Geriatr.

[70] French C, Burden S, Stanmore E (2024). Digital intervention (Keep-On-Keep-Up Nutrition) to improve nutrition in older adults: protocol for a feasibility randomized controlled trial. JMIR Res Protoc.

[71] French C, Burden S, Tang Y, Stanmore E (2025). A person-centred approach to further develop a digital tool (KOKU-Nut) by developing a nutrition game for older adults living in the community. BMC Geriatr.

[72] Happe L, Sgraja M, Hein A, Diekmann R (2022). Iterative development and applicability of a tablet-based e-coach for older adults in rehabilitation units to improve nutrition and physical activity: usability study. JMIR Hum Factors.

[73] Foerster M, Timper M, Puhlemann R, Quinten V, Diekmann R (2026). A digital assistive system for maintaining nutrition and mobility in older adults: usability and feasibility findings from a pilot study. JMIR Form Res.

[74] Förster M, Happe L, Quinten V, Diekmann R (2026). Development and iterative optimization of an independently usable assistance system to assess, maintain and improve the nutritional and mobility status of older adults: an iterative usability study. BMC Geriatr.

[75] Jung H, Demiris G, Tarczy-Hornoch P, Zachry M (2021). A novel food record app for dietary assessments among older adults with type 2 diabetes: development and usability study. JMIR Form Res.

[76] Liu YC, Chen CH, Lin YS (2020). Design and usability evaluation of mobile voice-added food reporting for elderly people: randomized controlled trial. JMIR mHealth uHealth.

[77] Zenun Franco R, Fallaize R, Lovegrove JA, Hwang F (2018). Online dietary intake assessment using a graphical food frequency app (eNutri): usability metrics from the EatWellUK study. PLoS One.

[78] Bosoni P, Gasparini G, Pe S, Larizza C, Quaglini S, Lanzola G (2026). Usability of mobile health apps from the patient perspective: a systematic review. Stud Health Technol Inform.

[79] Sharma V, Martinez MM, Sosa E (2025). Implementation fidelity of a digital nutrition education intervention program for community-dwelling older adults. J Nutr Educ Behav.

